# Broad-Spectrum Antimicrobial Activity of Oftasecur and Visuprime Ophthalmic Solutions

**DOI:** 10.3390/microorganisms11020503

**Published:** 2023-02-17

**Authors:** Federica Dell’Annunziata, Maria Vittoria Morone, Marco Gioia, Ferdinando Cione, Massimiliano Galdiero, Nicola Rosa, Gianluigi Franci, Maddalena De Bernardo, Veronica Folliero

**Affiliations:** 1 Department of Experimental Medicine, University of Campania Luigi Vanvitelli, 80138 Naples, Italy; 2 Department of Medicine, Surgery and Dentistry “Scuola Medica Salernitana”, University of Salerno, 84081 Baronissi, Italy; 3 Clinica Pathology and Microbiology Unit, San Giovanni di Dio e Ruggi D’Aragona University Hospital, 84126 Salerno, Italy

**Keywords:** antibacterial activity, antiviral potential, eye drops, Oftasecur, Visuprime, HSV-1, Gram-positive bacteria, Gram-negative bacteria

## Abstract

Due to the wide etiology of conjunctivitis, the expensive and time-consuming diagnosis requires new therapeutic strategies with broad-spectrum antimicrobial activity and nonselective mechanisms of action. In this context, eye drops could provide an alternative to conventional antimicrobial therapies. Here, we compare the antibacterial and antiviral activity of Oftasecur and Visuprime, commercially available ophthalmic solutions. Cytotoxicity assay was performed on Vero CCL-81 cells by 3-(4,5-dimethylthiazol-2-yl)-2,5-diphenyl-tetrazolium bromide (MTT) test. Antibacterial efficacy was evaluated on *Staphylococcus aureus*, *Staphylococcus epidermidis*, *Pseudomonas aeruginosa*, *Escherichia coli,* and *Klebsiella pneumoniae* by disk diffusion, broth microdilution methods, and time-killing tests. Furthermore, the antiviral activity against HSV-1 was estimated by co-treatment, cell and viral pretreatment and post-treatment, via plaque reduction assay, fluorescence assessment (GFP-engineered HSV-1), and real-time PCR. After 24 h of exposure, Oftasecur and Visuprime showed a volume-inducing 50% of cytotoxicity of 125 and 15.8 μL, respectively Oftasecur and Visuprime induced 90% antibacterial activity in response to mean volume of 10.0 and 4.4 µL for Gram-positive and Gram-negative strains, respectively. Oftasecur exerted bactericidal action on both bacterial populations, while Visuprime was bacteriostatic on Gram-negative strains and slightly bactericidal on Gram-positive bacteria. A major impact on infectivity occurred by exposure of viral particles to the ophthalmic solutions. In detail, 50% of inhibition was verified by exposing the viral particles to 3.12 and 0.84 μL of Oftasecur and Visuprime, respectively, for 1 h. The reduction of the fluorescence and the expression of the viral genes confirmed the recorded antiviral activity. Due to their high antimicrobial efficiency, Oftasecur and Visuprime could represent a valid empirical strategy for the treatment of conjunctivitis.

## 1. Introduction

Conjunctivitis represents a frequently encountered disorder in ophthalmology clinics around the world [1]. It involves inflammation and chemosis, associated with congestion of the blood vessels, secretions, and pain [2].

This disease is disseminated worldwide, with direct and indirect socioeconomic impacts, and represents the leading cause of visual morbidity. Conjunctivitis is a condition that affects more than 2% of the world’s population of any age, demographic, or socioeconomic class [1]. Over 80% of cases of conjunctivitis are diagnosed by non-ophthalmologists, resulting in a negative impact on the health system, due to clinic visits and prescriptions of unsuitable drugs [3]. In 2021, conjunctivitis management cost USD 4.55 billion and is projected to exceed USD 6.31 billion by 2030 [4]. This disease can be classified according to chronicity, the seriousness of the disorder, the implication of affected tissues, and etiology [5]. Regarding chronicity, conjunctivitis can be classified as acute, lasting up to four weeks, and chronic, with a duration of more than four weeks [6].

Conjunctivitis can be highly symptomatic, associated with rich mucopurulent secretions [7]. Additionally, inflammation can involve the lid margins and cornea, causing blepharoconjunctivitis and keratoconjunctivitis, respectively [8,9]. Conjunctivitis causes can include noninfectious and infectious conditions. Allergies and toxins are among the most common noninfectious causes of conjunctivitis [10]. About 80% of all cases of conjunctivitis are of an infectious nature [11]. Viruses are the leading cause of infectious conjunctivitis, followed by bacteria, although fungi and parasites may be involved in the progress of infection [12]. The microorganisms that cause conjunctivitis can be acquired by direct contact with hands and objects contaminated by the etiological agent, aerosol transmission, and vertically from mother to child [13,14,15].

The ocular district represents one of the main sites of viral replication and access for the spread in the extraocular regions [16,17]. This event is mainly paired with adenoviruses, picornaviruses such as coxsackievirus A24 and enterovirus 70, and herpesviruses, including herpes simplex virus type 1 and 2, Varicella-zoster virus, and Epstein–Barr virus, cytomegalovirus, herpes simplex virus 6 and 7, and Kaposi’s sarcoma herpesvirus [18,19]. As for bacterial conjunctivitis, Gram-positives account for 70% of ocular isolates, including *Staphylococcus aureus (S. aureus)*, *Staphylococcus epidermidis (S. epidermidis)*, and different species of Streptococci. In contrast, Gram-negative bacteria are responsible for 25% of disease cases, involving *Pseudomonas aeruginosa (P. aeruginosa)*, *Escherichia coli (E. coli)*, *Haemophilus influenzae*, *Neisseria gonorrhoeae*, and *Klebsiella pneumonia (K. pneumoniae)* [20,21]. Establishing the conjunctivitis etiology based on the patient’s symptomatology can be deceptive. The detection and identification of microorganisms causing conjunctivitis are essential for the prescription of effective and targeted therapy [22]. The laboratory tests used to identify the viral causative agent mainly involve polymerase chain reaction (PCR), but it is not widely used outside of hospital facilities [23]. Indeed, the microbiological diagnosis of bacterial conjunctivitis involves bacterial cultures which require approximately 48 h to identify the bacterial species and establish the antibiotic susceptibility profile [24]. Considering that diagnostic tests are unavailable and lengthy, empirical treatment is usually administered based on the clinician’s experience. Empirical treatment often proves ineffective due to the increase in drug-resistant strains [25,26]. Therefore, stricter requirements, conscious use of antibiotics and antiviral drugs, and novel therapeutic strategies in the sector of ophthalmology are essential.

In this context, antiseptic eye drops could provide an effective option to conventional therapies [27,28]. There are several reports of evidence of the low propensity of eye drops to trigger the development of resistance due to the lack of a defined action mechanism, unlike antibiotics and antiviral drugs. Moreover, the wide range of action makes them ideal broad-spectrum strategies [29]. The commonly used eye drops in ophthalmology are povidone-iodine (PVP-I) and chlorhexidine (CHX) [30]. Both exhibit effective antimicrobial activity against Gram-positive, Gram-negative, intracellular bacteria, fungi, viruses, and Acanthamoeba [12]. Numerous studies have proved that PVP-I acts on a broader spectrum of microorganisms, unlike CHX [31]. Moreover, no cases of resistance to PVP-I have been documented; conversely, microorganisms resistant to CHX have been identified [32]. Therefore, new investigations of commercially available ophthalmic solutions with broad-spectrum antimicrobial activity are needed.

The present study evaluated the eye drops Visuprime and Oftasecur for their antimicrobial potential. Oftasecur Ocular Spray ^®^ (OFFHEALTH Spa, Florence, Italy) is a recent commercial liposomal composition containing Biosecur, hypromellose, phospholipids S80, boric acid, sodium tetraborate decahydrate, chloride of sodium, and distilled water. This formulation has a protective and soothing function for the outer part of the eye and gives relief in the case of eye and eyelid irritation. Visuprime is a sterile, isotonic, and buffered lubricating ophthalmic solution with physiological pH based on Poloxamer 407, Polyquaternary 133, and disodium EDTA. The presence of poloxamer 407 creates a protective mechanical–physical barrier which lubricates and stabilizes the tear film, reducing tear evaporation and the risk of microbial contamination. Currently, a double-blind clinical study is underway to investigate the efficacy of Visuprime to reduce the conjunctival bacterial load in patients undergoing anti-VEGF injection (US Clinical Trials Registry: Clinical Trial NCT05677685). The comparison of the antimicrobial action of the two formulations could allow the identification of the right strategy to eradicate bacterial and viral conjunctivitis.

## 2. Materials and Methods

### 2.1. Ophthalmic Solutions

Visuprime and Oftasecur are commercially available eye drops used to protect and soothe the ocular surface. The compositions of the ophthalmic solutions are shown in Table 1.

### 2.2. Bacteria and Growth Conditions

The test bacterial strains were acquired from the American Type Culture Collection (ATCC). *S. aureus* (ATCC 6538)*, S. epidermidis* (ATCC 12228)*, P. aeruginosa* (ATCC 13388)*, E. coli* (ATCC 11229), *and K. pneumoniae* (ATCC 10031) were plated on Mueller Hinton (MH) agar (Oxoid, Hampshire, MA, USA) at 37 °C in aerobic condition overnight. Thereafter, the bacterial preinoculate was prepared by inoculating colonies of each bacterial strain in MH broth and then incubating at 37 °C overnight. The latter was resuspended in fresh broth until the exponential phase was reached. The inoculum was serially diluted to achieve the bacterial concentration of 5 × 10^5^ colony-forming units/mL (CFU/mL), required for the assays.

### 2.3. Cell Culture Conditions

The cell line derived from the renal epithelium of the African green monkey (*Cercopithecus aethiops*) (VERO ATCC CCL-81, Manassas, VA, USA) was used for cytotoxicity and antiviral assays. The cell line was cultured in Dulbecco’s Modified Eagle Medium (DMEM) with 4.5 g/L glucose (Gibco Life Technologies, Paisley, UK), supplemented with 2 mM L-glutamine (Gibco Life Technologies, Paisley, UK), 100 IU/mL penicillin–streptomycin solution (Gibco Life Technologies, Paisley, UK), and 10% Fetal Bovine Serum (FBS) (Gibco Life Technologies, Paisley, UK). For the tests, cells were plated in 96- and 24-well plates, in final volumes of 0.2 and 0.5 mL, respectively. The plates were incubated at 37 °C, with 5% CO_2_ in a humid environment.

### 2.4. Viruses and Propagation

The useful viruses were herpes simplex virus type-1 SC16 (HSV-1) containing an lacZ gene under the control of the cytomegalovirus IE-1 promoter and HSV-1 containing the Green Fluorescent Protein reporter inserted in the gene coding for the VP22 protein (HSV-1-GFP). Viral propagation was conducted by infecting the confluent CCL-81 Vero cell monolayer with HSV-1 at a multiplicity of infection (MOI) of 0.01, for 1 h at 37 °C. Subsequently, the nonpenetrated virus was removed and cells were incubated in DMEM supplemented with 10% FBS until a cytopathic effect developed. The virus particles were released from the infected cells through three freezing cycles. Subsequently, the lysates were centrifuged at 2500× *g* for 10 min and the supernatants were titrated by plaque reduction assay.

### 2.5. Cell Cytotoxicity Test

Cytotoxicity assays were performed by the 3-[4,5-dimethylthiazol-2-yl]-2,5 diphenyl tetrazolium bromide (MTT) method. Vero CCL-81 cells were plated in 96-well plates with a density of 2 × 10^4^ cells/well for a final volume of 0.2 mL. Oftasecur and Visuprime were serially diluted twice in a complete medium (200–1.56 µL). The growth medium was removed and cell monolayers were exposed to 200 µL of each dilution at different times (0.5, 2, and 24 h). Furthermore, DMSO and cells grown with the 1× phosphate-buffered saline (1× PBS) used to dilute the ophthalmic solutions constituted the positive (CTRL+) and negative (CTRL−) controls, respectively. Cell viability was assessed by adding 100 µL of the MTT solution (5 mg/mL) to the cell monolayers for 3 h. After incubation, the medium was removed and DMSO (100 µL) was used to dissolve the formazan salts. The cytotoxicity rates were obtained by measuring the absorbance at 570 nm using a microplate reader (Tecan life science, Männedorf, SW).

### 2.6. Disk Diffusion Test

The potential antibacterial activity of ophthalmic solutions was evaluated by the Kirby–Bauer disc diffusion method. Briefly, for each bacterial strain, the suspension at the density of 0.5 McFarland was uniformly plated on the surface of MH agar (Oxoid, Basingstoke, Hampshire, MA, USA). Thereafter, a paper disk (6.5 mm) was imbibed with 30 μL of eye drops and deposited on the agar plate. Piperacillin disc (30 μg) represented CTRL+. The plates were incubated at 37 °C overnight and the diameters of the zones of inhibition were measured and expressed as mm ± SD.

### 2.7. Antibacterial Susceptibility Assays

The antibacterial activity assays were conducted using the broth microdilution method. Oftasecur and Visuprime were serially diluted 1:2 in MH-broth (100, 50, 25, 12.5, 6.25, and 3.13 μL) and a constant volume of 100 μL was added to each well. Then, 100 uL of *S. aureus*, *S. epidermidis*, *P. aeruginosa*, *E. coli,* and *K. pneumoniae* at a density of 1 × 10^6^ CFU/mL were added and exposed to the ophthalmic solutions for 20 h. Vancomycin (10.4 μg/mL) constituted the CTRL+ for Gram-positive bacteria, Ampicillin (20 μg/mL) for *E. coli* and *K. pneumoniae*, while meropenem (40 μg/mL) represented the CTRL+ for *P. aeruginosa*. On the other hand, untreated bacteria posed the CTRL-. The turbidity was achieved using a microplate reader (Tecan, Männedorf, Swiss).

### 2.8. Bacterial Killing Kinetic Tests

The bacterial kinetics of action of ophthalmic solutions were evaluated by time-killing assays against *E. coli* and *S. aureus*, as reference strains of Gram-negative and -positive, respectively. Oftasecur (12.5–6.25 μL for *E. coli;* 6.25–3.12 μL for *S. aureus*) and Visuprime (3.12–1.56 μL for *E. coli;* 6.25–3.12 μL for *S. aureus*) were prepared for a final volume of 2 mL/tube. Untreated and antibiotic-treated bacterial suspensions constituted CTRL− and CTRL+, respectively. Then, a bacterial inoculum of 1 × 10^6^ CFU/mL was added to each test tube, obtaining a final density of 5 × 10^5^ CFU/mL, and incubated at 37 °C for up to 20 h. Aliquots of 100 μL were collected at 0, 3, 6, 9, and 20 h and serially diluted in MH broth. The dilutions were plated on MH agar, incubated at 37 °C overnight, and arising colonies were counted to determine the number of CFU/mL.

### 2.9. Plaque Assay

The effect of Oftasecur and Visuprime on HSV-1 infection was evaluated by co-treatment, cell and viral pretreatment and post-treatment via plaque reduction assays in infected cells. In the co-treatment assay, the eye drops at the selected volumes and the viral suspension at a density of 2 × 10^3^ plaque-forming units/mL (PFU/mL) were simultaneously inoculated on the cell monolayer in DMEM without FBS for 1 h at 37 °C. In cell pre-treatment, cell monolayers were first exposed to ophthalmic solutions for 1 h at 37 °C and then infected with the viral suspension at 2 × 10^3^ PFU/mL in DMEM without FBS for 1 h at 37 °C. In virus pretreatment, the viral suspension at 2 × 10^4^ PFU/mL was exposed to formulations for 1–10–30 min and 1 h, diluted 1:10 in DMEM without FBS, and used to infect cell monolayers, for 1 h. Lastly, in post-treatment, cells were first infected with the viral suspension at the density of 2 × 10^3^ PFU/mL in DMEM without FBS for 1 h at 37 °C; then, they were washed and treated with the formulations at the selected volumes for 1 h. Regarding CTRL+, melittin (5 µM) was applied in the co-treatment and virus pretreatment, dextran-sulfate (1 µM) in cell pretreatment, and aciclovir (5 µM) in post-treatment, whereas uninfected cells constituted CTRL−. After the viral adsorption time, the cell monolayer was washed twice with 1 × PBS and covered with a culture medium supplemented with 5% carboxymethylcellulose. After 48 h, Vero CCL-81 cells were fixed with 4% formaldehyde (Sigma-Aldrich, St. Louis, MO, USA) and stained with 0.5% crystal violet (Sigma-Aldrich, St. Louis, MO, USA). The plaques were counted and related to the coincident ones at the CTRL− to obtain the percentage of viral inhibition [31]. Lastly, the virus pretreatment assay was also confirmed using GFP-engineered HSV-1, exposing to 12.5–3.12 µL of Oftasecur and 6.25–1.56 µL of Visuprime for 1 h, prior to cell infection. Bright-field and fluorescent images were acquired by the Nikon ECLIPSE Ti2-U fluorescence microscope (Nikon Europe B.V., Amsterdam, The Netherlands) after 48 h of exposure. The fluorescence intensity was gained by Cytation 5 plate reader (Cytation 5, BioTek, Milan, Italy).

### 2.10. Real-Time Molecular Analysis

The results obtained in the viral pretreatment assays were confirmed by evaluating the expression of the UL27 and UL54 genes by a quantitative polymerase chain reaction (qPCR). Infected treated and untreated cells were harvested and subjected to RNA extraction by TRIzol (Thermo Fisher, Waltham, MA, USA). Then, total RNA was quantified via nanodrop (NanoDrop 2000, Thermo Fisher Scientific, Waltham, MA, USA) and 1 ug was exploited to obtain cDNA by reverse transcription reaction based on the instructions of the SensiFAST ™ cDNA Synthesis Kit (Meridian Bioscience, Washington, DC, USA). The qPCR was set up with 0.3 μM of each primer, 1 × BrightGreen qPCR MasterMix (abm, San Francisco, CA, USA), and 100 ng of cDNA. It was carried out in Thermal Cycler UNO96 (VWR International, Radnor, PA, USA) according to the amplification program: denaturation at 95 °C for 15 s, annealing at 60 °C for 20 s, and extension at 72 °C for 15 s (40 cycles). Target threshold cycle (Ct) values were subjected to normalization, exploiting the expression of glyceraldehyde 3-phosphate dehydrogenase (GAPDH). Data are shown according to 2^−ΔΔCt^ values.

### 2.11. Statistical Analyses

Assays were executed in biological triplicate and reported as mean ± standard deviation (SD). The volumes of eye drops associated with 90 and 50% antibacterial activity, 50% cytotoxicity and 50% antiviral activity were obtained from the dose–response curve via nonlinear regression analysis using the Graph Pad software Prism 9.0 (San Diego, CA, USA). The significance of the differences of the samples treated with the CTRL− was evaluated by one-way analysis of variance (ANOVA) with Dunnett’s test as post hoc, exploiting the Graph Pad Prism 9.0 software (San Diego, CA, USA). *p*-value < 0.05 defined significant data.

## 3. Results

### 3.1. Cytotoxic Activity

**Figure 1:**
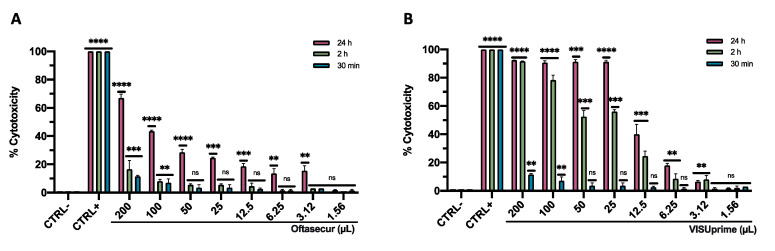
(**A**) Oftasecur cytotoxicity on Vero CCL-81 cell line after 0.5 (***: *p*-value = 0.0012, **: *p*-value = 0.0021, ns: *p*-value > 0.88), 2 (***: *p*-value < 0.0010, **: *p*-value < 0.0018, ns: *p*-value > 0.76) and 24 h (****: *p*-value < 0.0001, ***: *p*-value < 0.0015, **: *p*-value < 0.0023). (**B**) Visuprime cytotoxicity on Vero CCL-81 cell line after 0.5 (**: *p*-value < 0.0025, ns: *p*-value > 0.99), 2 (****: *p*-value < 0.0001, ***: *p*-value < 0.0010, **: *p*-value < 0.0018, ns: *p*-value > 0.76), and 24 h (****: *p*-value < 0.0001, ***: *p*-value < 0.0015, **: *p*-value < 0.0023, ns: *p*-value > 0.99).

The cytotoxicity of Oftasecur and Visuprime was investigated on Vero CCL-81 cells by the MTT method (Figure 1A,B). The cell monolayer was exposed to the ophthalmic solutions at volumes of 200, 100, 50, 25, 12.5, 6.25, 3.13, and 1.56 µL for 0.5, 2, and 24 h. DMSO induced 100% toxicity while a dose-dependent cytotoxic was registered for ophthalmic solutions, at all exposure times. In detail, after 0.5 h of treatment, volumes that induced 50% cytotoxicity were less than 200 μL for both formulations. This dose remained below 200 μL for Oftasecur, while it was reduced to 23.8 μL for Visuprime, after 2 h of exposure. The mortality rate was lower than 20% after 24 h of treatment, exhibiting volume-inducing 50% cytotoxicity of 125 and 15.8 μL for Oftasecur and Visuprime, respectively.

### 3.2. Antibacterial Activity

**Figure 2:**
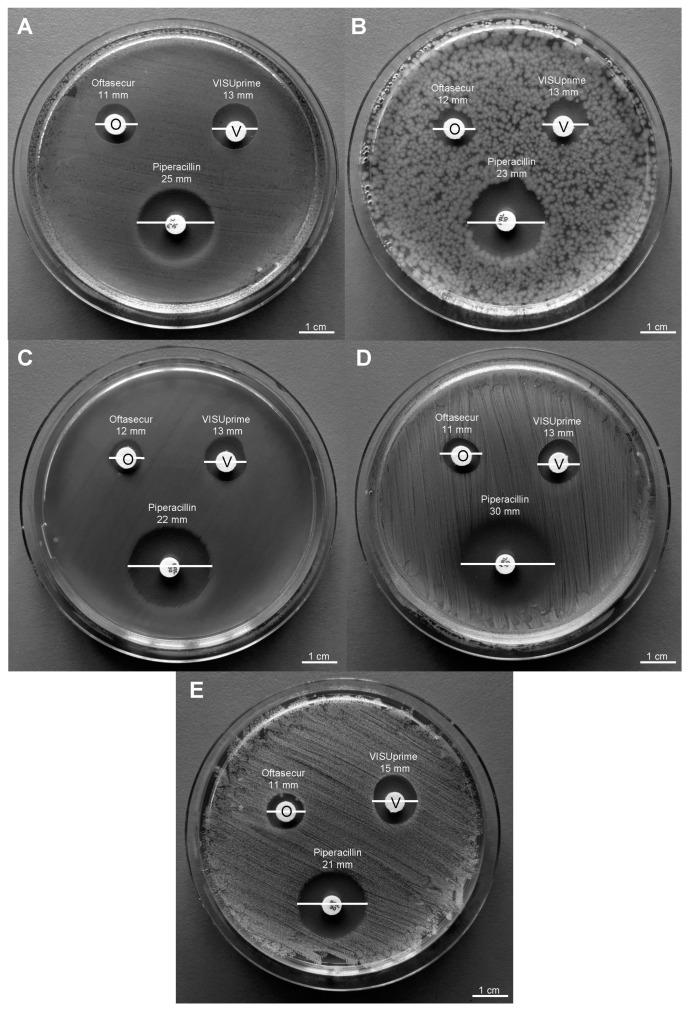
Oftasecur (O) and Visuprime (V) inhibition zone against *E. coli* (**A**), *K. pneumoniae* (**B**), *P. aeruginosa* (**C**), *S. aureus* (**D**), and *S. epidermidis* (**E**). Piperacillin (30 μg) was used as CTRL+.

**Figure 3:**
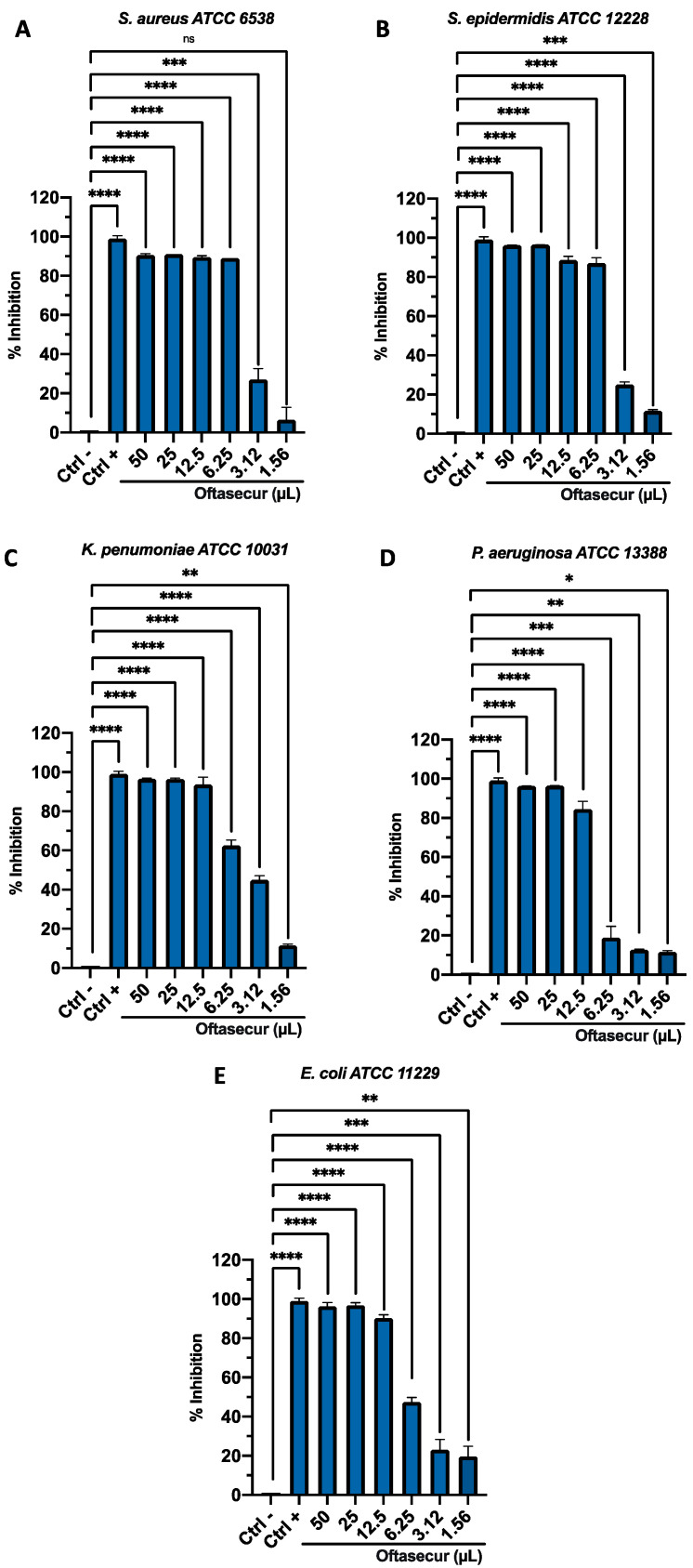
Antimicrobial activity of Oftasecur against (**A**) *S. aureus* (****: *p*-value < 0.0001, ***: *p*-value < 0.0014, ns: *p*-value > 0.71); (**B**) *S. epidermidis* (****: *p*-value < 0.0001, ***: *p*-value = 0.0009); (**C**) *K. pneumoniae* (****: *p*-value < 0.0001, **: *p*-value = 0.0012)*;* (**D**) *P. aeruginosa* (****: *p*-value < 0.0001, ***: *p*-value = 0.0008, **: *p*-value = 0.0016, *: *p*-value = 0.023)*;* (**E**) *E. coli* (****: *p*-value < 0.0001, ***: *p*-value = 0.0006, **: *p*-value = 0.0019).

**Figure 4:**
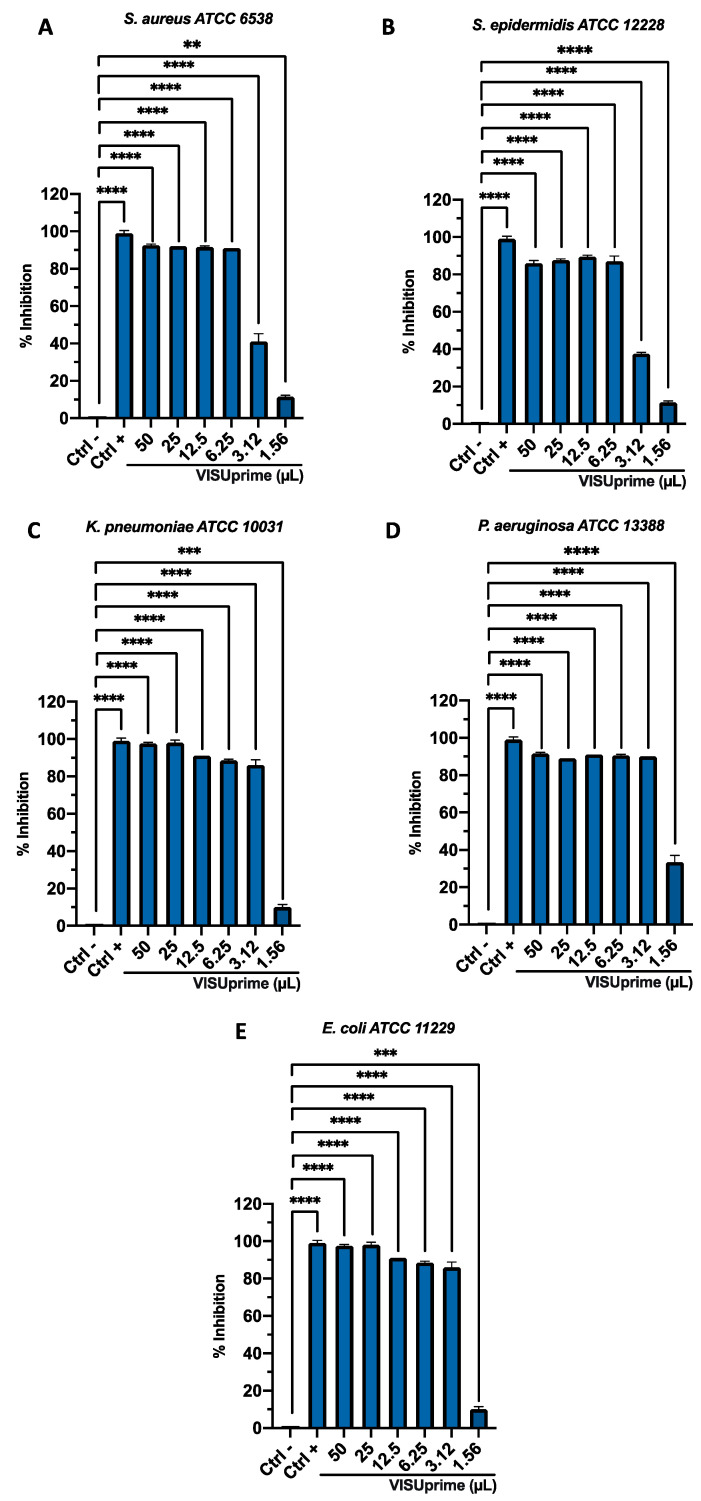
Antimicrobial activity of Visuprime against (**A**) *S. aureus* (****: *p*-value < 0.0001, **: *p*-value = 0.0013); (**B**) *S. epidermidis* (****: *p*-value < 0.0001)*;* (**C**) *K. pneumoniae* (****: *p*-value < 0.0001, ***: *p*-value = 0.0002); (**D**) *P. aeruginosa* (****: *p*-value < 0.0001); (**E**) *E. coli* (****: *p*-value < 0.0001, ***: *p*-value = 0.0006).

**Figure 5:**
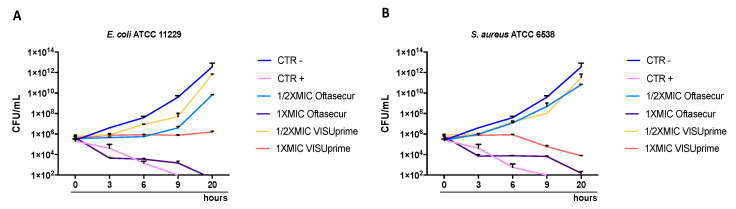
Oftasecur and Visuprime killing kinetics on (**A**) *E. coli* and (**B**) *S. aureus*.

The antibacterial potential of ophthalmic solutions was evaluated by Kirby–Bauer disc diffusion tests, broth microdilution method, and time-killing assays against Gram-positive and Gram-negative strains. All the tests performed proved the involvement of both formulations in altering bacterial growth. The halos of inhibition tricked by formulations were 11 ± 0.20, 12 ± 0.29, 12 ± 0.19, 11 ± 0.21, and 11 ± 0.34 mm for Oftasecur and 13 ± 0.13, 13 ± 0.21, 13 ± 0.27, 13 ± 0.31, and 15 ± 0.33 mm for Visuprime, related to *E. coli*, *K. pneumoniae, P. aeruginosa*, *S. aureus,* and *S. epidermidis*. Furthermore, Piperacillin induced a mean inhibition diameter of 24.2 ± 3.56 mm (Figure 2). By the broth microdilution assay, the Gram-positive and Gram-negative strains showed impaired viability compared to the untreated control. For Oftasecur, volumes inducing 50% antibacterial activity were 6.41, 4.14, 4.72, 8.93, and 4.24 μL for *E. coli*, *S. aureus*, *K. pneumoniae*, *P. aeruginosa,* and *S. epidermidis*, respectively; on the other side, volumes inducing 90% antibacterial activity were 12.95, 6.34, 10.10, 14.16, and 6.69 μL for the same strains (Figure 3A–E). After Visuprime exposure, volumes inducing 50% antibacterial activity were 2.42, 3.58, 2.51, 1.93, and 3.83 μL *E. coli*, *S. aureus*, *K. pneumoniae*, *P. aeruginosa,* and *S. epidermidis*, respectively; volumes inducing 90% antibacterial activity were 3.28, 5.93, 3.31, 3.14, and 6.52 μL for the aforementioned bacteria (Figure 4A–E). Time-killing assays documented the kinetics action of eye drops on selected bacterial strains. Visuprime at volume inducing 90% antibacterial activity did not alter the bacterial load of the Gram-negative strain, compared to that recorded at time 0 of the CTRL−. Oppositely, the same formulation acted as a mild bactericidal agent against the Gram-positive strain. In detail, starting from 6 h of exposure, a reduction of the bacterial load of 33.3 times compared to the initial bacterial load (CTRL−) occurred. Treatment with Visuprime at a dose inducing 50% antibacterial activity did not induce relevant changes in the growth curve relative to the CTRL−. Conversely, Oftasecur showed bactericidal effects on both bacterial classes. Specifically, a gradual reduction of the bacterial load up to 5.6 × 10^3^ and 1.7 × 10^3^ times for the Gram-negative and Gram-positive strains was recorded after 20 h (Figure 5).

### 3.3. Antiviral Activity

**Figure 6:**
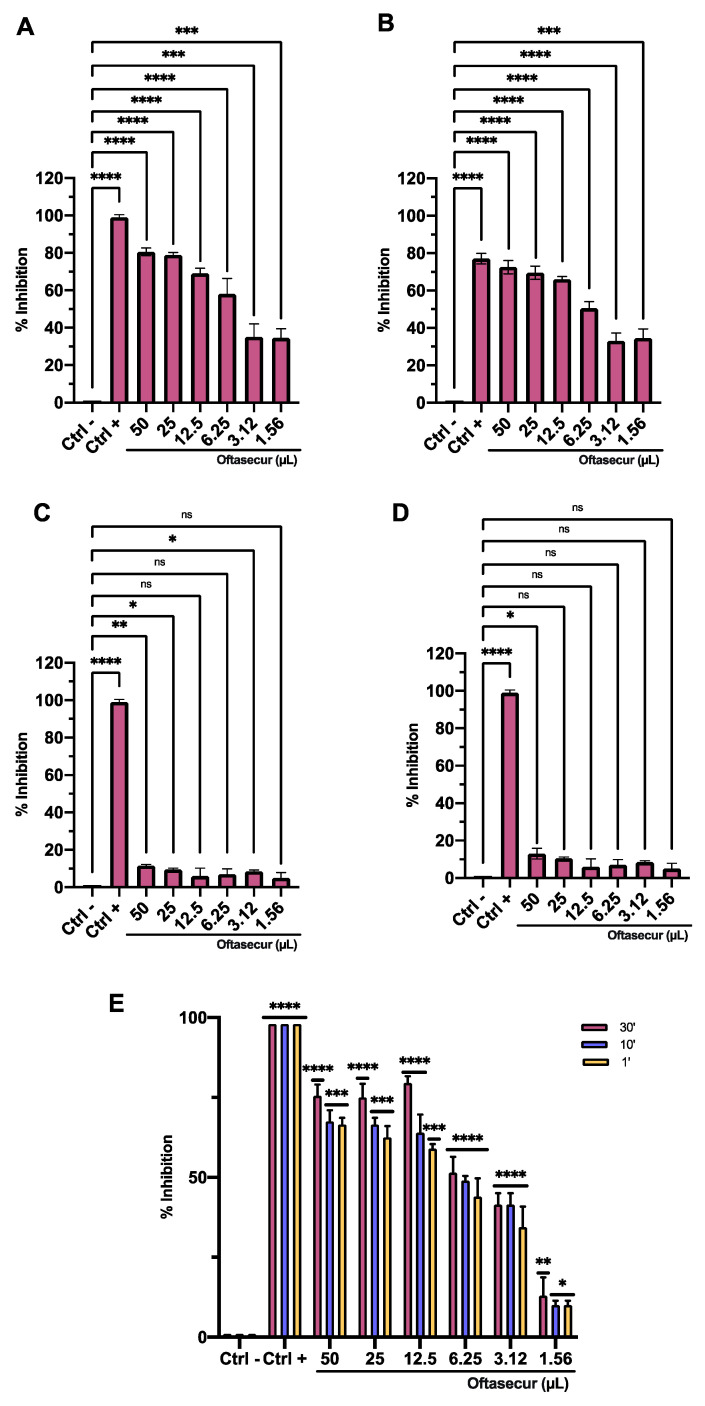
Antiviral activity of Oftasecur against HSV-1 in four plaque reduction assays: (**A**) co-treatment assay (****: *p*-value < 0.0001, ***: *p*-value = 0.0004); (**B**) pre-virus assay (****: *p*-value < 0.0001)*;* (**C**) cell pretreatment assay (****: *p*-value < 0.0001, **: *p*-value = 0.0063, *: *p*-value = 0.0208, ns: *p*-value > 0.1044)*;* (**D**) post-treatment assay (****: *p*-value < 0.0001, *: *p*-value = 0.0187, ns: *p*-value > 0.0600). (**E**) Pre-virus assay after 30, 10, and 1 min of exposure (****: *p*-value < 0.0001, ***: *p*-value = 0.0002, **: *p*-value = 0.0073; *: *p*-value = 0.039).

**Figure 7:**
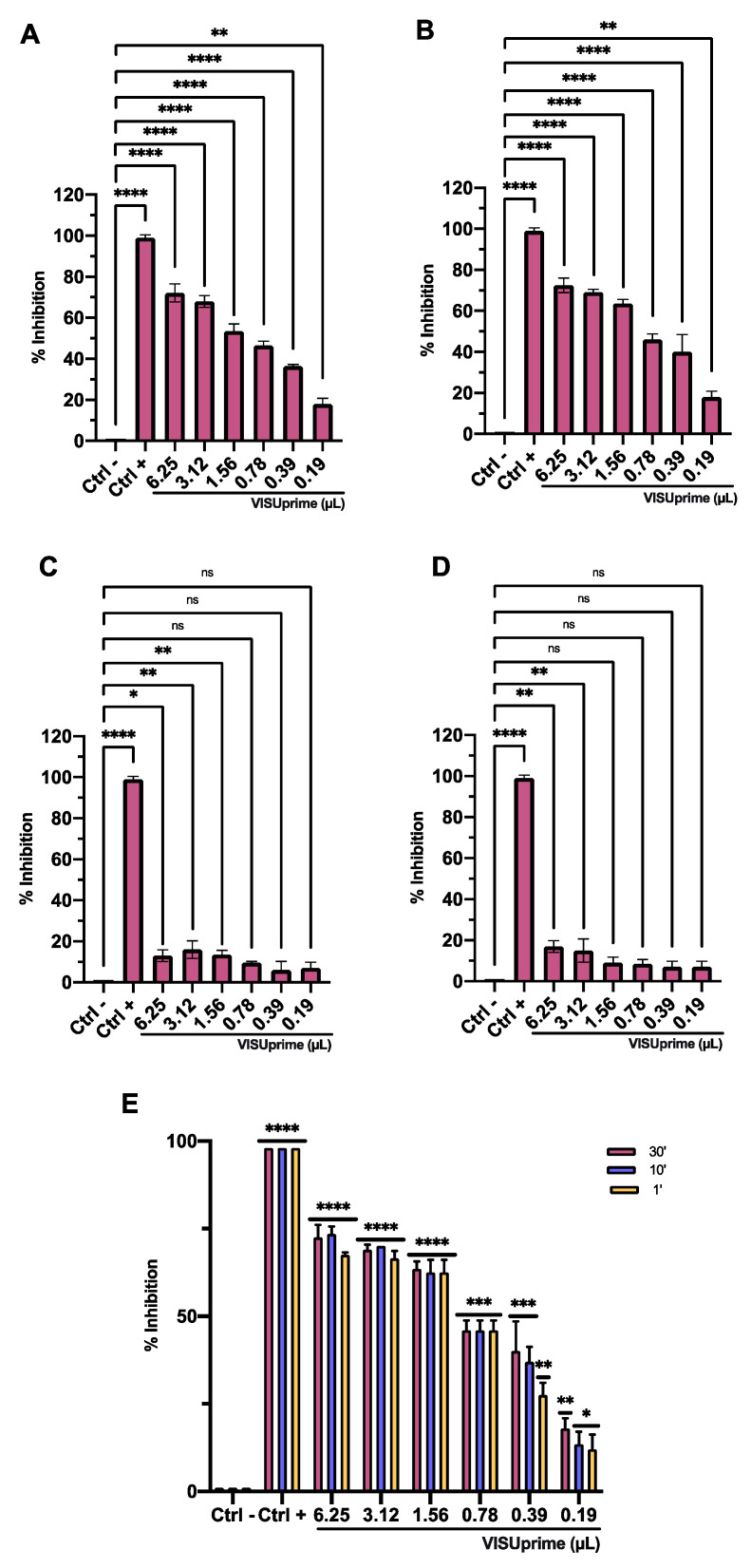
Antiviral activity of Visuprime against HSV-1 in four plaque reduction assays: (**A**) co-treatment assay (****: *p*-value < 0.0001, **: *p*-value = 0.0065); (**B**) pre-virus assay (****: *p*-value < 0.0001; **: *p*-value = 0.0086); (**C**) cell pretreatment assay (****: *p*-value < 0.0001, **: *p*-value = 0.0086, *: *p*-value = 0.0109, ns: *p*-value > 0.06280); (**D**) post-treatment assay (****: *p*-value < 0.0001, **: *p*-value = 0.0075, ns: *p*-value > 0.1163). (**E**) Pre-virus assay after 30, 10, and 1 min of exposure (****: *p*-value < 0.0001, ***: *p*-value = 0.0003, **: *p*-value = 0.0074; *: *p*-value = 0.069).

**Figure 8:**
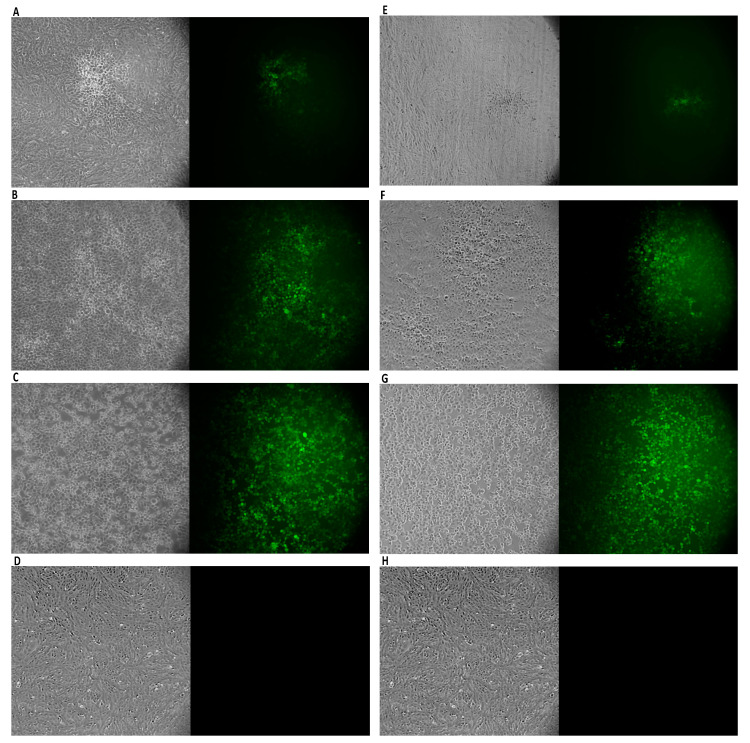
Antiviral activity of Oftasecur against GFP-engineered HSV-1. Plaques were viewed with bright-field and fluorescent microscopy: treatments at a 12.5 (**A**) and 3.12 (**B**) µL; CTRL−: cells infected with the virus (**C**); CTRL+: cells uninfected (**D**). Antiviral activity of Visuprime against GFP-engineered HSV-1. Plaques were viewed with bright-field and fluorescent microscopy: treatments at a 6.25 (**E**) and 1.56 (**F**) µL; CTRL−: cells infected with the virus (**G**); CTRL+: cells uninfected (**H**).

**Figure 9:**
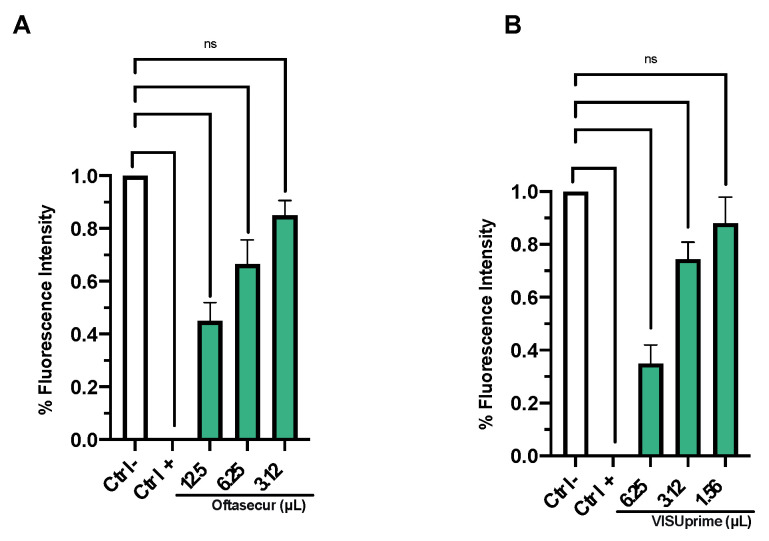
Virus pretreatment with Oftasecur (**A**) and Visuprime (**B**) against GFP-engineered HSV-1. The fluorescence signal was read at the excitation wavelength of 395 nm and the emission wavelength of 509 nm. (**A**): (****: *p*-value < 0.0001, ***: *p*-value = 0.0007, **: *p*-value = 0.0063; ns: *p*-value = 0.1300); (**B**): (****: *p*-value < 0.0001, ***: *p*-value = 0.0004, *: *p*-value = 0.0255; ns: *p*-value = 0.2724).

The antiviral potential of Oftasecur and Visuprime was evaluated against HSV-1 by plaque reduction assays. In the co-treatment test, both ophthalmic solutions showed a significant ability to interfere with the viral infection, reporting volumes inducing 50% antiviral activity of 5.3 and 1.57 μL for Oftasecur and Visuprime, respectively. Thus, cell and virus pretreatment assays and post-treatment assays were performed to investigate the infection target. A greater impact was verified by exposing the viral suspension to ophthalmic formulations. In particular, 50% of infection inhibition occurred, incubating the viral suspension to 3.12 and 0.84 μL for Oftasecur and Visuprime, respectively. Conversely, no impairment was recorded when cells were treated with ophthalmic solutions before and after viral infection. To evaluate the kinetics of action of eye drops, the virus pretreatment was performed at shorter times of exposure (30, 10, and 1 min) at 37 °C. Setting the threshold to 50% viral inhibition, Visuprime was effective against HSV-1 up to 1.56 μL for all time points selected. On the other hand, Oftasecur showed a half-maximal inhibitory at 12.5 μL at the same exposure times (Figure 6 and Figure 7). Further demonstration of antiviral efficacy was obtained by fluorescence microscopy (Figure 8 and Figure 9). In detail, a virus pretreatment assay was performed using GFP-engineered HSV-1, which dyes infected cells fluorescent green. For the assay, two volumes (one functional and one inactive) were selected and the images were obtained in bright-field and fluorescent. The results obtained showed an inhibitory effect for both ophthalmic solutions at the highest amount used (12.5 and 6.25 μL for Oftasecur and Visuprime, respectively). In contrast, the cytopathic effect was significantly increased at 3.12 and 1.56 μL, demonstrated by a strong fluorescence signal, comparable to the virus control (CTRL−) (Figure 8). A similar assay was performed to quantify the intensity of fluorescence emitted. Then, after 48 h of exposure to GFP-engineered HSV-1, the fluorescent signal was read using the Cytation 5 Reader, at an excitation wavelength of 395 nm and an emission wavelength of 509 nm. The results identified a dose-dependent increase in fluorescence signal. Specifically, at 12.5 and 6.25 μL, the lowest intensity recorded was 0.45 and 0.37 for Oftasecur and Visuprime, respectively (Figure 9A,B). Overall, these findings were consistent with previous results obtained through plaque reduction tests.

### 3.4. Molecular Investigation of UL27 and UL54

**Figure 10:**
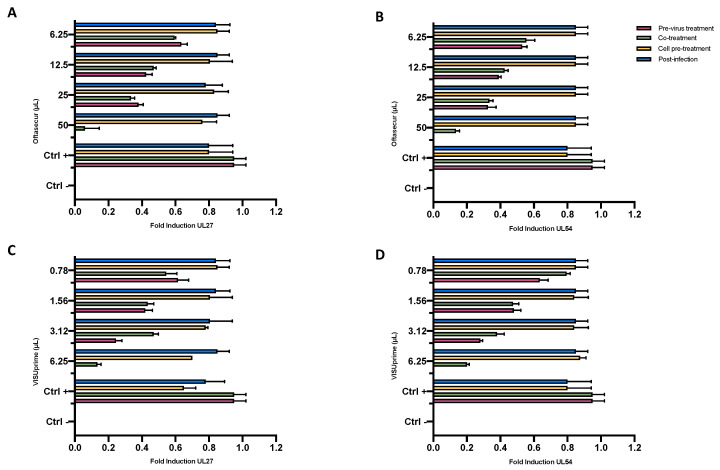
Quantification of mRNA levels of UL27 and UL54 expressed in HSV-1 infected Vero CCL-81 cells after exposure to Oftasecur (**A**,**B**) and Visuprime (**C**,**D**) in different stages of viral infection. Data are presented as the ratio of reference genes (GAPDH) to target genes.

Confirmation of the results obtained by the previous assays was conducted by molecular investigation. The genes analyzed were UL54, which encodes the immediate–early multifunctional protein essential for HSV infection, and UL27, the late gene encoding the protein envelope structural glycoprotein B (gB). For the molecular analysis, all treatments were performed under the same experimental conditions described above. qPCR showed variations in gene expression depending on the dose of eye drops and the type of assay performed. In detail, the expression of UL27 and UL54 was completely inhibited in the virus pretreatment with Oftasecur at 50 µL and increased in a dose-dependent manner up to 6.25 µL, reaching a fold induction of 0.7. In co-treatment, a similar trend occurred, while in cell pretreatment and post-treatment the gene expression of both was comparable to the control virus (Figure 10A,B). Regarding the inhibition induced by Visuprime, at the highest amount tested (6.25 µL) for both genes, a total block of expression was recorded in the virus pretreatment, while a fold induction of 0.2 and 0.27 was detected for UL27 and UL54 in the co-treatment. Conversely, at the same condition, no alteration of gene expression occurred in the other two assays (Figure 10C,D). Therefore, the results obtained suggested that Oftasecur and Visuprime played an important inhibitory role in the impairment of HSV-1 replication steps.

## 4. Discussion

In this study, the antimicrobial efficacy of Visuprime and Oftasecur was investigated. Both ophthalmic solutions were commercially available to soothe and protect the surface of the eye. In the ophthalmology field, antiseptics are becoming a relevant therapeutic approach due to their broad-spectrum activity for nonselective mechanisms of action [33,34]. Little evidence reports the antimicrobial potential of Visuprime and Oftasecur.

In this context, we tested both eye drops against *S. aureus, S. epidermidis, P. aeruginosa, E. coli, K. pneumoniae,* and HSV-1. The Kirby–Bauer disk diffusion assay permitted the evaluation of the eye drops’ preliminary antibacterial activity. A mean zone of inhibition of 11.4 ± 0.55 and 13.4 ± 0.89 mm was induced in response to the treatment of Gram-positive and Gram-negative strains with Oftasecur and Visuprime, respectively. The best antibacterial efficacy of Visuprime was confirmed by broth microdilution and time-killing tests. This agent inhibited the growth of Gram-positive and Gram-negative strains by 90%, with an average dose of 4.4 µL. In contrast, 10.1 µL of Oftasecur was required to impair bacterial growth. Consistent with our results, a preliminary study conducted by Mencucci and colleagues reported the antimicrobial effect of Oftasecur against *S. aureus*, *S. epidermidis*, *S. pyogenes*, *E. coli*, *P. aeruginosa,* and *C. albicans*, recording a bactericidal effect and fungicide against the microorganisms analyzed [33]. Otherwise, no studies to date have investigated the antimicrobial effect of Visuprime.

Based on our research, the antibacterial activity of ophthalmic solutions is mainly due to the presence of Biosecur and polyquaternium-based (PQ) compounds in Oftasecur and Visuprime, respectively. Biosecur antiseptic consists of citrus flavonoids with broad antibacterial potential. Cormier et al. proved that Biosecur completely eradicated *Vibrio vulnificus* by exposing the bacterial culture (10^5^ CFU) to 2% Biosecur [35]. Additionally, de Nova et al. showed its antibacterial action, reporting MIC values in the range from 16 to 128 μg/mL versus *Campylobacter coli, Campylobacter jejuni, E. coli, Salmonella enterica ssp. Enterica, Clostridium difficile, Clostridium. perfringens,* and *S. aureus* [36]. Several studies investigated the antibacterial activity of PQ. Codling et al. reported a significant antibacterial efficacy of PQ-1 against *P. aeruginosa*, *Serratia marcescens,* and *S. aureus*, inducing potassium ion loss [37]. Moreover, Ziklo et al. proved that PQ-80 completely impaired the growth of *P. aeruginosa*, *S. aureus,* and *E. coli*, upon exposure with 62.5, 4, and 31.25 ppm, respectively [38]. Time-killing data reported the different kinetics of ophthalmic formulations on Gram-positive and Gram-negative bacterial strains. Oftasecur was depicted as a bactericidal agent, reducing the bacterial load after 3 h of exposure, for both bacterial populations. In contrast, Visuprime did not compromise the bacterial load of the Gram-negative compared to the initial one, while it reduced the density of the Gram-positive after 6 h of treatment. The different behavior of the two ophthalmic solutions could be due to the composition of the formulations and the different structures of the bacterial cell wall. The flavonoids, a constituent of Oftasecur, act by compromising the structure and function of cell membranes and, consequently, bacterial vitality [39,40]. However, PQ compounds are cationic polymers and their action is expressed through interaction with the cell membrane, causing its rupture and leak of the cell contents. The thick peptidoglycan layer of the wall of Gram-positive bacteria protects cells from the action of PQ compounds, explaining the higher volumes inducing 90% antibacterial activity values obtained for Gram-positive strains.

The antiviral potential of Oftasecur and Visuprime was evaluated against HSV-1 by plaque reduction assays. A greater impact was exhibited when the viral particles were exposed to the ophthalmic formulations, recording volumes inducing 50% antiviral activity at 3.12 and 0.84 μL for Oftasecur and Visuprime, respectively. From viral kinetic assays, it emerged that both eye drops were capable of compromising the viral structure even after 1 min of exposure, thus demonstrating a rapid time action. The reduction of fluorescence signals and the early and late gene modulation expression proved the anti-herpes efficacy of ophthalmic formulations. The lethal activity of Visuprime could be linked to the structural and functional alteration of the envelope, causing the nonrecognition of the host cell and the consequent release of the viral genome [41]. Various evidences proved the antiviral activity of PQ compounds. Rohan et al. showed that 100 µL of Vagisil, a PQ-32-based product, inhibited HIV replication by 95% [42]. Moreover, Macinga et al. showed that adding PQ-37 to ethanol-based disinfectant increased its antiviral action against norovirus [43]. Polyphenol-based compounds also were described for their antiviral properties. Torky et al. proved that the polyphenolic moiety of hibiscus extracts exhibited significant anti-herpes efficacy, interfering with virus–cell recognition [44]. Moreover, Yi et al. demonstrated that luteolin exhibited a high affinity for the S protein of the coronavirus, preventing its entry into human cells [45]. This evidence proved that the phenolic component acted by masking the viral receptors, blocking the cell–virus interaction, while the lipidic agents exercised a virucidal action by altering the structure/function of the envelope. For instance, liposomes have been shown to interact with the steroid component of the viral envelope, impairing HIV, HCV, and HBV infection [46]. Moreover, several studies showed that 50 µg/mL of oleic acid destroys the viral envelope, preventing the infectious event [46]. In light of these considerations, the lipidic nature of Visuprime could justify the better antiviral activity against the enveloped virus.

Compared with the antibiotics commonly used for the treatment and prophylaxis of eye infections, Oftasecur and Visuprime could represent a more advantageous strategy. To date, fluoroquinolones, aminoglycosides, sulfonamides, and chloramphenicol are the most effective drugs against Gram-positive and -negative bacteria; however, the increase in MDR ocular pathogens is limiting their use [32]. A study conducted by Antibiotic Resistance Monitoring in Ocular Microorganisms showed that *S aureus* (34.9%) and CoNS isolates (49.3%) were resistant to methicillin with concomitant resistance of *S. aureus* to macrolides (18.34%), fluoroquinolones (22.61%), and aminoglycosides (18.29%). Antibiotic resistance among *S. pneumoniae* isolates was highest for azithromycin (36.3%), while *P. aeruginosa* and *H. influenzae* showed low resistance [47]. In addition to the MDR phenomenon, numerous adverse effects were recorded following their use. Allergic and photosensitivity reactions may occur; aminoglycosides cause irreversible and cumulative ototoxicity over time, which limits treatments to a short duration and is nonrepetitive [48]; fluoroquinolones can have serious muscular and cardiovascular side effects, hepatic cytolysis, and digestive dysbiosis [49]. Furthermore, the administration of some drugs is contraindicated in pregnant women and children under 14 years of age [50]. Otherwise, there is no specific treatment for viral eye infections; indeed, cold compresses, artificial tears, and topical antihistamines are indicated to relieve symptoms. For viral conjunctivitis caused by HSV or zoster virus, ganciclovir gel or acyclovir ointment are the only drugs currently available [51]. Considering this evidence, the use of eye drops with broad-spectrum antimicrobial action could represent a solution for difficult-to-manage eye infections, minimizing side effects.

Contextually, due to their broad-spectrum antimicrobial activity associated with safe use, Oftasecur and Visuprime could be considered valid alternatives for the treatment of conjunctivitis of viral and bacterial etiology. Future studies will be aimed at evaluating the antimicrobial activity of the aforementioned eye drops versus clinically isolated viral and bacterial strains, highlighting their mechanism of action.

## Figures and Tables

**Table 1:** Compositions of the ophthalmic solutions.

Ophthalmic Solutions	Composition (100 mL)
Visuprime	PQ133 (100 mg), poloxamer 407 (4.500 mg), disodium EDTA (100 mg), isotonic buffered solution.
Oftasecur	Biosecur (2 g), hypromellose (0.15 g), phospholipids S80, boric acid, sodium tetraborate decahydrate, sodium chloride, distilled water.

## Data Availability

Not applicable.
